# Orofacial myofunctional changes and risk of dysphagia in older adults in the frailty process: a methodological study

**DOI:** 10.1590/2317-1782/e20250043en

**Published:** 2026-01-26

**Authors:** Thaline Moura de Oliveira, Stela Maris Aguiar Lemos, Aline Mansueto Mourão

**Affiliations:** 1 Mestrado Acadêmico em Ciências Fonoaudiológicas, Universidade Federal de Minas Gerais – UFMG - Belo Horizonte (MG), Brasil.; 2 Departamento de Fonoaudiologia, Faculdade de Medicina, Universidade Federal de Minas Gerais – UFMG - Belo Horizonte (MG), Brasil.

**Keywords:** Mastication, Deglutition, Stomatognathic System, Deglutition Disorders, Aged, Frailty

## Abstract

**Purpose:**

to verify the agreement between the evaluation of orofacial myofunctional aspects and the risk of dysphagia in older people in the frailty process.

**Methods:**

methodological study with 100 individuals from a referral center for older people, assessed using the Orofacial Myofunctional Evaluation Protocol with Scores for Older People (OMES-O) and the Oropharyngeal Dysphagia Screening in Older Adults (RaDI). Information regarding participants’ chewing disorders, age, and years of education was also collected. ROC curve analysis was performed, and sensitivity, specificity, positive and negative predictive values were generated from this analysis.

**Results:**

the OMES-O presented a median of 236.5 points. Most participants were not at risk of dysphagia. The comparison between OMES-O and RaDI indicated no difference in the score of orofacial myofunctional aspects between individuals at risk and not at risk of dysphagia. Moreover, OMES-O was not associated with chewing alterations, age, or years of education.

**Conclusion:**

The evaluation of orofacial myofunctional aspects could not indicate the risk of oropharyngeal dysphagia in older adults in the frailty process.

## INTRODUCTION

Aging leads to changes in the anatomy and physiology of the stomatognathic system, involving bone loss, decreased salivary flow, changes in smell and taste, tooth loss, and decreased strength and mass of orofacial and cervical muscles^(1,2)^. Studies indicate that decreased oral function is likely to progress to malnutrition and affect physical function and socialization during aging^(3,4)^. Thus, orofacial myofunctional changes can lead older people to perform differently in the swallowing function^(5)^, causing presbyphagia or dysphagia.

The biomechanics of swallowing is a continuous process with interdependent phases. Especially in older adults, the preparatory and oral phases can be described by different masticatory and swallowing parameters, such as number of cycles, laterality (simultaneous/alternating bilateral or preferential unilateral), amplitude of mandibular displacements, contraction of the periorbicular and chin muscles, anterior tongue projection, head movement, noisy swallowing, among other aspects of lip, tongue, and cheek mobility during the eating process^(4,6)^.

The Orofacial Myofunctional Evaluation Protocol with Scores for Older People (OMES-O)^(7)^ assesses orofacial myofunctional aspects, considering the specificities of older adults. It identifies, classifies, and grades changes in the components and functions of the stomatognathic system caused by aging, including chewing and swallowing, from the perspective of clinical diagnosis of orofacial myofunctional disorders. There are still no validated protocols that aim to clinically evaluate and diagnose oropharyngeal dysphagia in older adults, considering their anatomical and physiological specificities. However, there are dysphagia screening protocols for older adults, such as the Oropharyngeal Dysphagia Screening in Older Adults (RaDI)^(8)^, which screens dysphagia signs and symptoms through a self-reported questionnaire, informing whether there is a risk of this condition.

The literature indicates that dysphagia and frailty are highly prevalent among older adults^(9)^, and that frail older adults are at increased risk of oropharyngeal dysphagia^(10)^. Hence, given the specificities of care for older people regarding changes in the structures and functions of the stomatognathic system caused by aging and the impact of these changes on the diagnosis of dysphagia, it is necessary to investigate whether a protocol for assessing myofunctional structures and functions, such as OMES-O, provides information on dysphagia screening in older adults in the frailty process, assessing their risk of older adults with orofacial myofunctional changes having dysphagia. Thus, this study aimed to verify the correlation between orofacial myofunctional assessment in older adults in the frailty process and their risk of oropharyngeal dysphagia.

## METHODS

Methodological study with 100 individuals from a referral center for older people, who were evaluated using two instruments: myofunctional aspects through OMES-O and the risk of dysphagia through RaDI.

OMES-O was used to evaluate the appearance and posture of stomatognathic system components, inspect their oral cavity, test their lip, tongue, jaw, and cheek mobility, and analyze their breathing pattern and spontaneous speech. Chewing and swallowing were assessed by offering solid food (a sandwich cookie, as recommended in the literature) and liquid food (200 ml of filtered water in a glass). The participant was instructed to ingest as usual^(7)^. The maximum protocol score is 250 points; scores between 202 and 250 indicate the absence of orofacial myofunctional disorders.

The RaDI is a self-reported questionnaire that aims to identify symptoms of oropharyngeal dysphagia in asymptomatic individuals or those with initial symptoms. It consists of nine questions, and the score ranges from 0 to 18 points^(8)^. The risk of dysphagia is confirmed in scores higher than 3 points^(11)^.

Information regarding the participants’ chewing disorders, age, and years of education was also collected. Questions related to chewing were assessed using the Screening for Masticatory Disorders in Older Adults (SMDOA), which aims to detect chewing disorders in these individuals. It has nine questions, with scores ranging from 0 to 18 points; higher scores indicate a greater risk of chewing disorders^(12)^. Data regarding age and education were obtained by reading the physical and/or electronic medical records and interviewing the patient and their companions. All instruments were administered in a single session by the same professional from October 2022 to October 2023.

Study participants were selected using the following criteria: aged 60 years or older, classified as at risk of frailty or frail using the Clinical-Functional Vulnerability Index-20 (IVCF-20)^(13)^, being followed by the multidisciplinary team at the referral center, and having updated medical records with previous history, comorbidities, diagnosis, exams, and clinical follow-up. The study excluded older adults who presented drowsiness and/or an inadequate level of consciousness for completion of the study (Glasgow Coma Scale < 12)^(14)^, severe cognitive impairment (Clinical Dementia Rating - CDR 3)^(15)^, or other diagnoses that compromised comprehension (such as comprehension aphasia or severe/profound hearing loss), and used an alternative feeding route.

It is important to note that the IVCF-20, used as a sample selection tool, covers multidimensional aspects of older adults' health status with 20 questions. It has a maximum score of 40 points; the higher the score, the greater the risk of clinical and functional vulnerability. Based on the results, individuals are categorized as: robust (0 to 6 points); at risk of frailty (7 to 14 points); and frail (15 to 40 points)^(13)^.

The ROC curve was analyzed to determine whether the OMES-O provided information on the risk of dysphagia in older adults, using the RaDI as the reference standard. Due to the small number of participants with abnormal OMES-O scores (n = 7), only those without a clinical diagnosis of orofacial myofunctional disorders were included in the analysis based on the cutoff. The ROC curve identified a cutoff in the OMES-O that can indicate the risk of dysphagia based on RaDI findings. Sensitivity, specificity, and positive and negative predictive values ​​were generated from this analysis. Statistical Package for the Social Sciences (SPSS), version 25.0, was used for data entry, processing, and analysis.

Descriptive data analysis consisted of the frequency distribution of categorical variables and the analysis of measures of central tendency and dispersion of continuous variables. The Shapiro-Wilk and Kolmogorov-Smirnov tests were applied and indicated that continuous variables were not normally distributed. Therefore, these variables are presented as medians and quartiles. The Mann-Whitney test was used to compare orofacial myofunctional aspects with the risk of dysphagia in older adults. A simple linear regression model was proposed to explain the response variable of orofacial myofunctional aspects (in its continuous form), based on the variables of risk of dysphagia in older adults, screening for masticatory disorders in older adults, age, and years of education. Bivariate analyses (the individual relationship of the explanatory with the response variables) were initially presented for this model. Because all variables are quantitative, the univariate analysis applied was the Spearman correlation test. The multivariate analysis considered continuous and categorical variables with a p-value ≤ 0.20.

The research was approved by the Research Ethics Committee under approval number 6.059.301, and all participants agreed to participate in the research by signing an informed consent form.

## RESULTS

The participants’ median age was 84 years (min = 64, max = 98), with a mean of 82 years (SD = 7.0); 62% were female. They had a median of 4 years of education (min = 0, max = 12), with a mean of 4 years (SD = 2.6). The total SMDOA score had a median of 7 points, indicating changes in the masticatory function. The OMES-O had a median of 236.5 points, indicating the absence of orofacial myofunctional disorders (100%). Most participants (78%) were not at risk of dysphagia (Table 1).

**Table 1:** Classification of the Clinical-Functional Vulnerability Index-20 and descriptive analysis of sociodemographic and clinical variables of orofacial motor function and risk of dysphagia (N = 100)

**IVCF-20**		**N**		**%**	
At risk of frailty (7-14)		27		27.0	
Frail (15-40)		73		73.0	
**Categorical variables**		**N**		**%**	
Sex					
Males		38		38.0	
Females		62		62.0	
RaDI					
0-3 (no changes)		78		78.0	
≥4 (initial diagnosis of dysphagia)		22		22.0	
**Continuous variables**	**Mean (SD)**	**Q_1_**	**Median**		**Q_3_**
Age	82(7.0)	78	84		87
Years of education	4(2.6)	3	4		4
Total SMDOA score	7(3.4)	4.3	7		10
Total OMES-O score	234(10.4)	228	236.5		242

**Caption:** IVCF-20 = Classification of the Clinical-Functional Vulnerability Index-20; N = number of participants; RaDI = Oropharyngeal Dysphagia Screening in Older Adults; Q_1_ = quartile one; Q_3_ = quartile three; SMDOA = Screening for Masticatory Disorders in Older Adults; OMES-O = Orofacial Myofunctional Evaluation Protocol with Scores for Older People; SD = standard deviation

The comparison of OMES-O and RaDI indicated no difference in orofacial myofunctional scores between individuals at risk and not at risk of dysphagia (p = 0.482) (Table 2). Moreover, OMES-O was not associated with changes in chewing (according to SMDOA) (p = 0.129), age (p = 0.312), or years of education (p = 0.947).

**Table 2:** Comparison and correlation between orofacial myofunctional aspects and the risk of dysphagia in older people (N = 100)

	**RaDI**		**p-value** *
	**No changes** (n = 78)	**With changes** (n = 22)	
**OMES-O**	237 (228 - 243)	235.5 (227.8 - 241.3)	0.482
Median (Q_1_-Q_3_)			
Spearman's Correlation Coefficient	0.070		
p-value	0.490		

* Mann-Whitney test

**Caption:** N = number of participants; RaDI = Oropharyngeal Dysphagia Screening in Older Adults; OMES-O = Orofacial Myofunctional Evaluation Protocol with Scores for Older People; Q_1_ = quartile one; Q_3_ = quartile three

**Figure 1:**
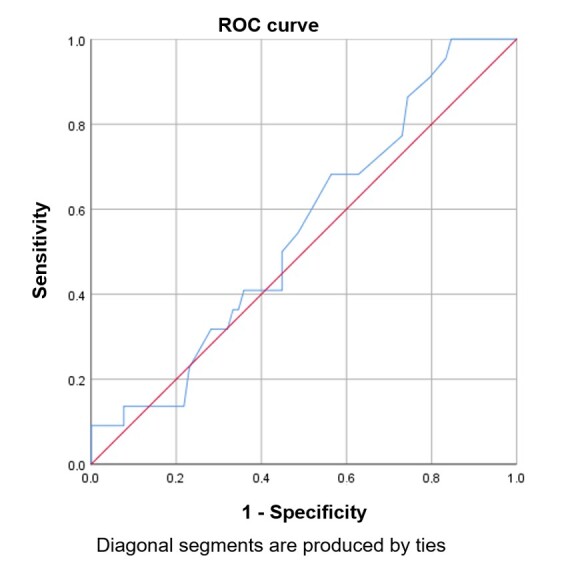
Relationship between sensitivity and specificity of the Orofacial Myofunctional Evaluation Protocol with Scores for Older People (OMES-O)

In Figure 1, the area under the ROC curve shows the total OMES-O score. The analysis was also not significant (rho = -0.153; p = 0.129), indicating that the OMES-O results in this sample were unable to indicate risk of dysphagia (p = 0.482). The area under the curve (i.e., the predictive capacity) was 54.9%. The sensitivity and specificity were 0.983 and 0.487, respectively. The positive predictive value was 0.245, and the negative predictive value was 0.809.

## DISCUSSION

The study demonstrated that the orofacial myofunctional assessment was unable to indicate the risk of oropharyngeal dysphagia in older people, highlighting the need to apply specific instruments to evaluate the biomechanics of swallowing.

A study^(16)^ applied the OMES-O to understand orofacial characteristics of functionally independent older adults, analyzing the association with age, sex, socioeconomic status, and dental status. It found that the oromyofacial system was within normal limits in most functionally independent older adults^(16)^. The present study, with older people in the frailty process, also demonstrated the absence of myofunctional disorders in most individuals, according to the cutoff established by the protocol authors. Thus, because it is a protocol specifically designed for myofunctional assessment in older adults, OMES-O considers most changes as part of senescence.

Changes in skeletal structures, orofacial and cervical muscles, oral mucosa, salivary glands, taste, and smell^(1,2)^ can affect chewing^(12)^ and swallowing functions^(4,6)^. A study^(10)^ used OMES-O to aid in the assessment of swallowing function in older adults. Aiming to estimate the prevalence and risk factors for oropharyngeal dysphagia in older adults hospitalized for trauma-orthopedic fractures, the authors applied the OMES-O to identify, classify, and grade the stomatognathic system components and functions and observed changes in mobility and masticatory performance, leading to restriction of solid consistencies in 57.6% of individuals^(10)^. It is worth noting that, unlike the present study, the aforementioned study was carried out with hospitalized older adults.

In this scenario, the RaDI identifies oropharyngeal dysphagia symptoms in asymptomatic older adults or those with initial symptoms. A study^(17)^ sought to relate nutritional risk and signs and symptoms of swallowing disorders reported by hospitalized older people, as well as correlate the total score of the Mini Nutritional Assessment (MNA) with the total number of signs and symptoms. The RaDI questions were used even before the protocol validation process was completed at the time of data collection, as it was considered, from a psychometric point of view, the most consistent instrument in Brazilian Portuguese at that time. There was no significant correlation between the total MNA score and the total number of signs and symptoms of swallowing disorders, but the mean total MNA score was lower in relation to those without complaints of choking^(17)^. While the present study analyzed the RaDI categorically, the authors analyzed the signs and symptoms of dysphagia continuously and observed that the total number of signs and symptoms of swallowing disorders ranged from zero to seven, and half of the sample reported at least one symptom. They also reported the absence of a validated instrument to investigate the signs and symptoms of swallowing disorders at the time of data collection^(16)^. Later, the authors of the RaDI found that this screening questionnaire can be a satisfactory screening tool to estimate the prevalence of oropharyngeal dysphagia in older people^(11)^.

Approximately a quarter of the participants in this study were at risk of dysphagia according to the RaDI. A systematic review with meta-analysis^(18)^ that estimated the prevalence of oropharyngeal dysphagia in adults in different healthcare settings found a 42% prevalence of oropharyngeal dysphagia in rehabilitation centers. However, the authors reported using data from only two studies in this setting^(18)^. Furthermore, that literature review included adults in general (over 18 years old), while the sample of the present study consisted of older adults in the frailty process, when the line between senescence (natural physiological transformations resulting from aging) and senility (changes that gradually cause a decline in the functioning of body systems) is more tenuous^(19)^. Therefore, further specialized studies are needed on oropharyngeal dysphagia, especially regarding older adults. These results also demonstrate the need to further investigate swallowing function and dysphagia signs and symptoms in older people, in addition to the clinical conditions and contextual factors involved.

Most participants in this study were classified as frail, meaning they were at increased risk of oropharyngeal dysphagia. However, 78% of participants were not at risk of dysphagia. One study found that more vulnerable older adults had greater difficulty perceiving illness and a lack of awareness of their overall bodily limitations^(10)^. Therefore, since the RaDI is a self-reported questionnaire, these findings may be explained by a possible difficulty in perceiving and identifying the signs and symptoms of oropharyngeal dysphagia among frail older adults.

Although OMES-O considers the specificities of senescence related to orofacial myofunctional structures and functions, it was unable to provide information on the risk of dysphagia in older adults in the frailty process. The diagnosis of dysphagia is based on both the safety and efficiency of swallowing biomechanics. However, it is known that oral myofunctional changes impact functional swallowing performance^(1)^, bringing adaptations to the eating process^(1,20)^, leading to presbyphagia, increasing the vulnerability of older adults, reducing their physiological reserve, and making them more susceptible to dysphagia^(1,21)^.

The lack of agreement between the two protocols may have been due to study limitations. Although this study was based on the historical series of older adults seen biannually at the referral center where the data were collected, it was necessary to perform a sample size calculation to stratify the groups "older adults at risk of frailty" and "frail older adults." Furthermore, there were no participants with myofunctional alterations in the sample, according to the OMES-O.

However, the study presents important advances in assessing the scope of the OMES-O, demonstrating that speech-language-hearing pathologists should be attentive to changes in swallowing function and the patients’ signs, symptoms, and complaints. Furthermore, the SMDOA and RaDI, because they screen specific functions, may be useful or complementary as warning signs. For future studies, we suggest comparing the two protocols in a larger number of participants at risk of frailty and including robust individuals with orofacial myofunctional alterations in the sample.

## CONCLUSION

The orofacial myofunctional assessment was unable to indicate the risk of oropharyngeal dysphagia in older adults in the frailty process. However, OMES-O provides relevant information regarding the structures and functions of orofacial motor function and should be used concomitantly with other instruments aimed at identifying changes in swallowing function in older adults.
