# Supplementary material for: Orofacial myofunctional changes and risk of dysphagia in older adults in the frailty process: a methodological study
Source: Codas. 2026 Jan 26;38(1):e20250043. doi: 10.1590/2317-1782/e20250043en (PMC12978334; doi:10.1590/2317-1782/e20250043en)
Supplement: Translation

# Translation: Modificações miofuncionais orofaciais e risco para disfagia em pessoas idosas em processo de fragilização: um estudo metodológico

**DOI:** 10.1590/2317-1782/e20250043pt

**Authors:** Thaline Moura de Oliveira, Stela Maris Aguiar Lemos, Aline Mansueto Mourão

**Affiliations:** 1 Mestrado Acadêmico em Ciências Fonoaudiológicas, Universidade Federal de Minas Gerais – UFMG - Belo Horizonte (MG), Brasil.; 2 Departamento de Fonoaudiologia, Faculdade de Medicina, Universidade Federal de Minas Gerais – UFMG - Belo Horizonte (MG), Brasil.

**Keywords:** Mastigação, Deglutição, Sistema Estomatognático, Transtornos de Deglutição, Idoso, Fragilidade

## Abstract

**Objetivo:**

verificar a concordância entre a avaliação dos aspectos miofuncionais orofaciais em pessoas idosas em processo de fragilização e o risco para disfagia.

**Método:**

estudo metodológico realizado com 100 indivíduos de um centro de referência à pessoa idosa, avaliados por meio da Avaliação Miofuncional com Escores para Idosos (AMIOFE-I) e pelo Rastreamento de Disfagia Orofaríngea em Idosos (RaDI). Também foram coletadas informações referentes às alterações de mastigação, à idade, e aos anos de estudo dos participantes. Foi realizada análise da curva ROC e a sensibilidade, a especificidade, o valor preditivo positivo e o valor preditivo negativo foram gerados a partir dessa análise.

**Resultados:**

o protocolo AMIOFE-I apresentou mediana de 236,5 pontos. Em relação à deglutição, a maioria dos participantes não apresentou risco para disfagia. A comparação dos protocolos AMIOFE-I e RaDI não indicou diferenças na pontuação dos aspectos miofuncionais orofaciais quando se comparam indivíduos sem e com risco para disfagia. Também não houve associação entre o AMIOFE-I e as alterações na mastigação, idade e anos de estudo.

**Conclusão:**

a avaliação dos aspectos miofuncionais orofaciais nas pessoas idosas em processo de fragilização não foi capaz de indicar a presença de risco para disfagia orofaríngea.

## INTRODUÇÃO

O envelhecimento leva a modificações na anatomia e na fisiologia do sistema estomatognático, envolvendo perda óssea, diminuição do fluxo salivar, alterações de olfato e paladar, perdas dentárias, diminuição da força e da massa da musculatura orofacial e cervical^(1,2)^. Estudos indicam que é provável que a diminuição da função oral progrida para a desnutrição, além de afetar a função física e a socialização durante o envelhecimento^(3,4)^. Assim, as modificações miofuncionais orofaciais podem levar a pessoa idosa a apresentar um desempenho diferenciado na função de deglutição^(5)^, ocasionando presbifagia ou disfagia.

A biomecânica da deglutição é um processo contínuo, sendo suas fases interdependentes. Especialmente na pessoa idosa, as fases preparatória e oral podem ser descritas por diferentes parâmetros mastigatórios e de deglutição, como número de ciclos, lateralidade (bilateral simultânea/alternada ou unilateral preferencial), amplitude dos deslocamentos mandibulares, contração da musculatura periorbicular e de mentual, projeção anterior da língua, movimentação de cabeça, deglutição ruidosa, dentre outros aspectos de mobilidade de lábios, língua e bochechas durante o processo alimentar^(4,6)^.

A Avaliação Miofuncional Orofacial com Escores para Idosos (AMIOFE-I)^(7)^ possibilita avaliar os aspectos miofuncionais orofaciais, considerando as especificidades da pessoa idosa. Este instrumento identifica, classifica e gradua as mudanças nos componentes e funções do sistema estomatognático ocasionadas pelo envelhecimento, incluindo a mastigação e a deglutição, sob a perspectiva de diagnóstico clínico de distúrbios miofuncionais orofaciais. Ainda não há protocolos validados que objetivem avaliar e diagnosticar clinicamente a disfagia orofaríngea no indivíduo idoso, considerando as especificidades anatomofisiológicas desta população. Contudo, há protocolos de rastreio da disfagia em pessoas idosas, como o Rastreamento de Disfagia Orofaríngea em Idosos (RaDI)^(8)^, que visa rastrear a presença de sinais e sintomas de disfagia por meio de um questionário autorreferido, informando se há risco para esta condição.

A literatura aponta que a disfagia e a fragilidade apresentam alta prevalência entre as pessoas idosas^(9)^ e as pessoas idosas frágeis apresentam risco aumentado para disfagia orofaríngea^(10)^. Desta forma, tendo em vista as especificidades da atenção à pessoa idosa em relação às modificações em estruturas e funções do sistema estomatognático ocasionadas pelo envelhecimento e os impactos dessas alterações no diagnóstico de disfagia, faz-se necessário investigar se um protocolo de avaliação das estruturas e funções miofuncionais, como o AMIOFE-I, possibilita trazer informações sobre o rastreamento para disfagia em pessoas idosas em processo de fragilização, verificando o risco de a pessoa idosa com modificações miofuncionais orofaciais ser disfágica. Assim, o objetivo do presente trabalho foi verificar a concordância entre a avaliação dos aspectos miofuncionais orofaciais em pessoas idosas em processo de fragilização e o risco para disfagia orofaríngea.

## MÉTODO

Estudo metodológico realizado com 100 indivíduos de um centro de referência à pessoa idosa, que foram avaliados usando dois instrumentos: os aspectos miofuncionais por meio do AMIOFE-I e o risco para disfagia, obtido pelo resultado do RaDI.

O protocolo AMIOFE-I permitiu a avaliação da aparência e postura dos componentes do sistema estomatognático, bem como a inspeção da cavidade oral, provas de mobilidade de lábios, língua, mandíbula e bochechas, análise do modo respiratório e análise da fala espontânea. A mastigação e deglutição foram avaliadas por meio da oferta de alimento sólido (um biscoito recheado, conforme preconizado na literatura) e líquido (200 ml de água filtrada no copo). O participante foi orientado a realizar a ingesta de maneira habitual^(7)^. A pontuação máxima do protocolo é 250 pontos, indicando entre 202 e 250 a ausência de distúrbios miofuncionais orofaciais.

O RaDI trata-se de um questionário autorreferido e visa identificar sintomas de disfagia orofaríngea em indivíduos assintomáticos ou com sintomas iniciais. É composto por nove perguntas e a pontuação varia entre zero e 18 pontos^(8)^. O risco para disfagia é confirmado em pontuações superiores a três pontos^(11)^.

Também foram coletadas informações referentes às alterações de mastigação, à idade, e aos anos de estudo dos participantes. As questões referentes à mastigação foram avaliadas pelo Rastreamento de Alterações Mastigatórias em Idosos (RAMI) que objetiva detectar alterações mastigatórias nesses indivíduos. Este instrumento é composto por nove perguntas, com pontuação que varia entre zero e 18 pontos, sendo que quanto maior a pontuação, maior o risco de alterações mastigatórias^(12)^. Os dados referentes à idade e à escolaridade foram obtidos por meio da leitura do prontuário físico e/ou eletrônico e entrevista com o paciente e seus acompanhantes. Todos os instrumentos foram aplicados em uma única sessão e pela mesma profissional, no período de outubro de 2022 a outubro de 2023.

Os indivíduos que participaram do estudo foram selecionados por meio dos seguintes critérios: idade igual ou acima de 60 anos, ser classificado como em risco de fragilização ou frágil por meio do Índice de Vulnerabilidade Clínico-Funcional-20 (IVCF-20)^(13)^; estar em acompanhamento com a equipe multidisciplinar do centro de referência e ter o prontuário atualizado com os registros de história pregressa, comorbidades, diagnóstico, exames e seguimento clínico. As pessoas idosas que apresentaram sonolência e/ou nível de consciência inadequado para realização do estudo (Escala de Coma de *Glasgow*< 12)^(14)^, comprometimento cognitivo grave (*Clinical Dementia Rating* - CDR 3)^(15)^ ou outros diagnósticos que comprometessem a compreensão (como afasia de compreensão ou perda auditiva grave/profunda) e uso de via alternativa de alimentação foram excluídos do estudo.

É importante mencionar que o IVCF-20, aplicado como instrumento de seleção da amostra, trata-se de um questionário que contempla aspectos multidimensionais da condição de saúde da pessoa idosa, sendo constituído por 20 questões. Apresenta uma pontuação máxima de 40 pontos e quanto mais alto o valor obtido, maior é o risco de vulnerabilidade clínico-funcional da pessoa idosa. A partir do resultado, os indivíduos são categorizados em: robustos (entre zero e seis pontos); em risco de fragilização (entre sete e 14 pontos) e frágeis (entre 15 e 40 pontos)^(13)^.

Para verificar se o AMIOFE-I possibilita trazer informações sobre o risco para disfagia em pessoas idosas foi analisada a curva ROC, tendo como padrão de referência o protocolo RaDI. Devido ao número reduzido de participantes com alteração no AMIOFE-I (n=7), de acordo com o ponto de corte, optou-se por incluir na análise somente os participantes sem diagnóstico clínico de distúrbios miofuncionais orofaciais. A curva ROC identifica um ponto de corte no protocolo AMIOFE-I que é capaz de identificar o risco para disfagia a partir das conclusões do RaDI. Sensibilidade, especificidade, valor preditivo positivo e negativo foram gerados a partir dessa análise. Para entrada, processamento e análise dos dados, utilizou-se o programa *Statistical Package for the Social Sciences* (SPSS), versão 25.0.

A análise descritiva dos dados constou da distribuição de frequência das variáveis categóricas e análise das medidas de tendência central e de dispersão das variáveis contínuas. Os testes de *Shapiro Wilk* e *Kolmogorov Smirnov* foram aplicados e indicaram que as variáveis contínuas não apresentaram distribuição normal. Dessa maneira, essas variáveis foram apresentadas em forma de mediana e quartis. O teste de *Mann Whitney* foi utilizado para comparação dos aspectos miofuncionais orofaciais pelo risco para disfagia nas pessoas idosas. Foi proposto um modelo de regressão linear simples para explicar a variável resposta de aspectos miofuncionais orofaciais (em sua forma contínua) a partir das variáveis de risco para disfagia nas pessoas idosas, rastreio de alterações mastigatórias em pessoas idosas, idade e anos de estudo. Para esse modelo, inicialmente foram apresentadas as análises bivariadas – relação individual das variáveis explicativas com a resposta. Por todas serem variáveis quantitativas, a univariada aplicada foi o teste de correlação de *Spearman*. Na análise multivariada, foram consideradas as variáveis contínuas e categóricas que apresentaram p-valor ≤0,20.

A pesquisa foi aprovada pelo Comitê de Ética em Pesquisas sob o número do parecer: 6.059.301 e todos os participantes manifestaram concordância em participar da pesquisa por meio da assinatura do Termo de Consentimento Livre e Esclarecido (TCLE).

## RESULTADOS

A mediana de idade dos participantes foi de 84 anos (mín = 64 e máx = 98), com média de 82 anos (DP = 7,0), sendo 62% do sexo feminino. O tempo de estudo apresentou mediana de quatro anos (mín = 0 e máx = 12), com média de quatro anos (DP = 2,6). A pontuação total do RAMI apresentou mediana de sete pontos, correspondendo à presença de alterações da função de mastigação. Já o protocolo AMIOFE-I apresentou mediana de 236,5 pontos, indicando ausência de distúrbios miofuncionais orofaciais (100%). Em relação à deglutição, a maioria dos participantes (78%) apresentou ausência de risco para disfagia (Tabela 1).

**Tabela 1:** Classificação do IVCF- 20 e análise descritiva das variáveis sociodemográficas e clínicas de motricidade orofacial e risco de disfagia (N=100)

**IVCF-20**		**N**		**%**	
Em risco de fragilização (7-14)		27		27,0	
Frágil (15-40)		73		73,0	
**Variáveis categóricas**		**N**		**%**	
Sexo					
Masculino		38		38,0	
Feminino		62		62,0	
Protocolo RaDI					
0-3 (sem alterações)		78		78,0	
≥4 (diagnóstico inicial de disfagia)		22		22,0	
**Variáveis contínuas**	**Média (DP)**	**Q1**	**Mediana**		**Q3**
Idade	82(7,0)	78	84		87
Anos de estudo	4(2,6)	3	4		4
Pontuação total no protocolo RAMI	7(3,4)	4,3	7		10
Pontuação total no protocolo AMIOFE-I	234(10,4)	228	236,5		242

**Legenda:** IVCF-20 = Índice de Vulnerabilidade Clínico Funcional-20; N = número de participantes; RaDI = Rastreamento de Disfagia Orofaríngea em Idosos; Q1 = Primeiro Quartil; Q3 = Terceiro Quartil; RAMI = Rastreamento de Alterações Mastigatórias em Idosos; AMIOFE-I = Avaliação Miofuncional Orofacial com Escores para Idosos; DP = desvio padrão

A comparação dos protocolos AMIOFE-I e RaDI indicou que não existe diferença na pontuação dos aspectos miofuncionais orofaciais quando se comparam indivíduos sem e com risco para disfagia (p = 0,482) (Tabela 2). Também não houve associação entre o AMIOFE-I e as alterações na mastigação (de acordo com o RAMI) (p = 0,129), a idade (p = 0,312) e os anos de estudo (p = 0,947).

**Tabela 2:** Comparação e correlação entre os aspectos miofuncionais orofaciais com o risco de disfagia em pessoas idosas (N = 100)

	**RaDI**		**Valor p** ^*^
	**Sem alterações** (n = 78)	**Com alterações** (n = 22)	
**AMIOFE-I**	237 (228 - 243)	235,5 (227,8 - 241,3)	0,482
Mediana (Q1-Q3)			
Coeficiente de Correlação de *Spearman*	0,070		
Valor p	0,490		

* Teste de *Mann Whitney*

**Legenda:** N = número de participantes; RaDI = Rastreamento de Disfagia Orofaríngea em Idosos; AMIOFE-I = Avaliação Miofuncional Orofacial com Escores para Idosos; Q1 = Primeiro Quartil; Q3 = Terceiro Quartil

Na Figura 1, pode-se observar na área sob a curva ROC a pontuação total no protocolo AMIOFE-I. A análise também não se mostrou significativa (rho = -0,153; p = 0,129), indicando que os resultados do protocolo AMIOFE-I encontrados nesta amostra não foram capazes de indicar risco para disfagia (p = 0,482). A área sob a curva, ou seja, a capacidade preditiva foi de 54,9%. A sensibilidade e a especificidade foram, respectivamente, 0,983 e 0,487. O valor preditivo positivo foi 0,245 e o valor preditivo negativo foi 0,809.

**Figura 1:** Relação entre a sensibilidade e especificidade do protocolo AMIOFE-I

## DISCUSSÃO

O estudo demonstrou que a avaliação dos aspectos miofuncionais orofaciais nas pessoas idosas não foi capaz de indicar o risco para disfagia orofaríngea nesta população, demonstrando a necessidade da aplicação de instrumentos específicos para a avaliação da biomecânica da deglutição.

Um estudo^(16)^ que aplicou o protocolo AMIOFE-I com o objetivo de conhecer as características orofaciais de idosos funcionalmente independentes, analisando a associação com idade, gênero, nível socioeconômico e estado dentário, verificou que o sistema oromiofacial encontrava-se dentro dos padrões de normalidade na maioria dos idosos funcionalmente independentes^(16)^. O presente estudo, realizado com pessoas idosas em processo de fragilização, também demonstrou ausência de distúrbios miofuncionais na maioria dos indivíduos, de acordo com a nota de corte estabelecida pelos autores do protocolo. Desta forma, é possível verificar que, por se tratar de um protocolo elaborado especificamente para a avaliação miofuncional em pessoas idosas, o AMIOFE-I considera grande parte das modificações encontradas como parte da senescência.

As modificações nas estruturas esqueléticas, na musculatura orofacial e cervical, na mucosa oral, nas glândulas salivares, no paladar e no olfato^(1,2)^, podem levar às alterações nas funções de mastigação^(12)^ e deglutição^(4,6)^. O protocolo AMIOFE-I foi utilizado em uma pesquisa^(10)^ como forma de auxiliar a avaliação da função de deglutição em pessoas idosas. Com o objetivo de estimar a prevalência e fatores de risco para disfagia orofaríngea em indivíduos idosos hospitalizados por fraturas traumato-ortopédicas, os autores aplicaram o protocolo AMIOFE-I para identificar, classificar e graduar os componentes e funções do sistema estomatognático e observaram alterações de mobilidade e desempenho mastigatório, levando à restrição de consistências sólidas em 57,6% dos indivíduos^(10)^. Vale destacar que, diferentemente do presente estudo, a pesquisa citada foi realizada com pessoas idosas hospitalizadas.

Neste cenário, o protocolo RaDI possibilita identificar sintomas de disfagia orofaríngea em pessoas idosas assintomáticas ou com sintomas iniciais. Um estudo^(17)^ buscou relacionar risco nutricional e sinais e sintomas de alterações da deglutição referidas por indivíduos idosos hospitalizados, assim como correlacionar o escore total da Mini Avaliação Nutricional (MAN) e o total de sinais e sintomas, utilizou as questões do RaDI antes mesmo da finalização do processo de validação do protocolo na época da coleta, devido a ele ter sido considerado, do ponto de vista psicométrico, o instrumento mais consistente em português brasileiro naquele momento. Não houve correlação significativa entre o escore total da MAN e o número total de sinais e sintomas de alterações da deglutição, porém a média do escore total da MAN foi mais baixa em relação àqueles sem queixa de engasgos^(17)^. Enquanto o presente estudo analisou o RaDI de forma categórica, os autores analisaram os sinais e sintomas de disfagia de forma contínua e observaram que o número total de sinais e sintomas de alterações da deglutição oscilou entre zero e sete e metade da amostra referiu pelo menos um sintoma. Além disso, foi relatada a ausência de um instrumento validado para investigar os sinais e sintomas de alterações da deglutição à época da coleta dos dados^(16)^. Posteriormente, os autores do RaDI verificaram que este questionário de rastreio pode ser uma ferramenta de triagem satisfatória para estimar a prevalência de disfagia orofaríngea em pessoas idosas^(11)^.

Aproximadamente um quarto dos participantes deste estudo apresentou risco para disfagia pelo RaDI. Uma revisão sistemática com metanálise^(18)^ que estimou a prevalência de disfagia orofaríngea em adultos em diferentes cenários de assistência à saúde, verificou prevalência de 42% de disfagia orofaríngea em centros de reabilitação. Porém, os autores relatam que foram utilizados dados de apenas dois estudos nesse cenário^(18)^. É importante salientar que a referida revisão de literatura incluiu a população adulta (acima de 18 anos), enquanto a amostra do presente estudo foi composta por pessoas idosas em processo de fragilização, em que a linha entre a senescência (transformações fisiológicas naturais decorridas do envelhecimento) e senilidade (alterações que de forma gradual ocasionam um declínio no funcionamento dos sistemas corporais) é mais tênue^(19)^. Deste modo, fica evidente a necessidade de mais estudos sobre a disfagia orofaríngea no componente especializado, especialmente em relação à população idosa. Ademais, estes resultados demonstram a necessidade de se investigar de forma mais aprofundada a função de deglutição em pessoas idosas, os sinais e sintomas de disfagia nessa população, além das condições clínicas e fatores contextuais envolvidos.

A maioria dos participantes deste estudo foi classificada como frágil, ou seja, com risco aumentado para disfagia orofaríngea. No entanto, 78% dos participantes apresentou ausência de risco para disfagia. Um estudo apontou que as pessoas idosas mais vulneráveis apresentavam maior dificuldade na percepção de doenças e na falta de consciência a respeito das limitações globais do seu organismo^(10)^. Desta forma, visto que o RaDI é um questionário autorreferido, esses achados podem ser explicados por uma possível dificuldade na percepção e identificação dos sinais e sintomas de disfagia orofaríngea por parte das pessoas idosas frágeis.

Apesar do protocolo AMIOFE-I considerar as especificidades da senescência relacionada às estruturas e funções miofuncionais orofaciais, não foi capaz de trazer informações sobre o risco para disfagia em pessoas idosas em processo de fragilização. O diagnóstico de disfagia é pautado tanto na segurança quanto na eficiência da biomecânica da deglutição. No entanto, sabe-se que as modificações oromiofuncionais impactam no desempenho funcional da deglutição^(1)^, trazendo adaptações no processo alimentar^(1,20)^ e levando a presbifagia, contribuindo, assim, para aumentar a vulnerabilidade do idoso e reduzir sua reserva fisiológica, tornando-os mais suscetíveis a disfagia^(1,21)^.

A ausência de concordância entre os dois protocolos pode ter sido em decorrência das limitações do estudo: embora o presente estudo tenha se baseado na série histórica de número de pessoas idosas atendidas semestralmente no centro de referência onde os dados foram coletados, faz-se necessário a realização do cálculo amostral para estratificação dos grupos “pessoa idosa em risco de fragilização” e “pessoa idosa frágil”. Além disso, houve ausência de participantes com alterações miofuncionais de acordo com o AMIOFE-I na amostra.

Contudo, o estudo demonstra avanços importantes ao verificar o alcance do protocolo AMIOFE-I, possibilitando constatar que o fonoaudiólogo deve estar atento às alterações na função de deglutição e aos sinais, sintomas e queixas apresentados pelo paciente. Além disso, os protocolos RAMI e RaDI, por se tratarem se rastreio de funções específicas, podem ser úteis ou complementares como sinais de alerta. Para estudos futuros, sugere-se a comparação dos dois protocolos em um número maior de participantes em risco de fragilização, bem como a inclusão de indivíduos robustos e com alterações miofuncionais orofaciais na amostra.

## CONCLUSÃO

A avaliação dos aspectos miofuncionais orofaciais nas pessoas idosas em processo de fragilização não foi capaz de indicar o risco para disfagia orofaríngea nesta população. Contudo, o protocolo AMIOFE-I permite informações relevantes quanto às estruturas e funções da motricidade orofacial e deve ser utilizado concomitantemente a outros instrumentos que visem identificar as alterações na função de deglutição em pessoas idosas.
